# Paneth cells as the origin of intestinal cancer in the context of inflammation

**DOI:** 10.21203/rs.3.rs-2458794/v1

**Published:** 2023-01-19

**Authors:** Mathijs P. Verhagen, Rosalie Joosten, Mark Schmitt, Andrea Sacchetti, Jiahn Choi, Niko Välimäki, Lauri A. Aaltonen, Leonard H. Augenlicht, Riccardo Fodde

**Affiliations:** 1 Department of Pathology, Erasmus University Medical Center, Rotterdam, The Netherlands; 2 Institute of Pharmacology, University of Marburg, Germany; 3 Department of Cell Biology, Albert Einstein College of Medicine, New York, U.S.A.; 4 Department of Medical and Clinical Genetics, University of Helsinki, Helsinki, Finland

## Abstract

Paneth cells (PCs), responsible for the secretion of antimicrobial peptides in the small intestine and for niche support to *Lgr5*^*+*^ crypt-base columnar stem cells (CBCs), have been shown to respond to inflammation by dedifferentiating into stem-like cells in order to sustain a regenerative response^1,2^. Therefore, PCs may represent the cells-of-origin of intestinal cancer in the context of inflammation. To test this hypothesis, we targeted *Apc, Kras,* and *Tp53* mutations in Paneth cells by Cre-Lox technology and modelled inflammation by dextran sodium sulfate (DSS) administration. PC-specific loss of *Apc* resulted in multiple small intestinal tumors, whereas *Kras* or *Tp53* mutations did not. Compound *Apc* and *Kras* mutations in PCs resulted in a striking increase in tumor multiplicity even in the absence of the inflammatory insult. By combining scRNAseq with lineage tracing to capture the conversion of PCs into *bona fide* tumor cells, we show that they progress through a “revival stem cell” (RSC) state characterized by high *Clusterin* (*Clu*) expression and *Yap1* signaling, reminiscent of what has been previously observed upon irradiation of the mouse digestive tract^3^. Accordingly, comparison of PC- and *Lgr5*-derived murine intestinal tumors revealed differences related to Wnt signaling and inflammatory pathways which match the dichotomy of CBCs and injury-induced RSCs^4^ between human sporadic colon cancers and those arising in the context of inflammatory bowel diseases. Last, we show that western-style dietary habits, known to trigger a low-grade inflammation throughout the intestinal tract, underlie the analogous dedifferentiation of Paneth cells and their acquisition of stem-like features.

Taken together, our results show that intestinal cancer arises in the context of inflammation through the dedifferentiation of committed secretory lineages such as Paneth cells and the activation of the revival stem cell state. As such, a true quiescent stem cell identity may be hidden in fully committed and postmitotic lineages which, upon inflammation, support the regenerative response by re-entering the cell cycle and dedifferentiating into RSCs. The chronic nature of the tissue insult in inflammatory bowel diseases and even in the context of western-style dietary habits is likely to result in the expansion of cell targets for tumor initiation and progression.

## Paneth cells are the origin of intestinal tumors in the context of inflammation and loss of *Apc.*

The origin of the vast majority of cancers is thought to reside in stem- or progenitor-like cells which satisfy the needs for active proliferation, self-renewal, and differentiation capacity. This was demonstrated in the intestine where loss-of-function mutations in the *Apc* tumor suppressor gene successfully initiate adenoma formation only when they occur in *Lgr5*^+^ CBCs. When the same *Apc* mutation is introduced in more committed and shorter-lived transit-amplifying cells, tumor formation is halted at early micro-adenoma stages^5^. However, next to this “bottom-up” scenario, additional “top-down” models of intestinal tumorigenesis have been proposed where more committed intestinal cells located at higher positions along the crypt-villus axis are likely to initiate cancer especially in the context of tissue injury and inflammation^6,7^. In the specific case of colon cancer, western style dietary habits and chronic inflammation, two of the major etiologic factors associated with increased risk for sporadic malignant disease in the digestive tract, are thought to induce specific cellular and molecular alterations of the intestinal epithelium which ultimately lead to the expansion of cell targets for tumor initiation and progression^6,8^. However, which specific cell lineages are capable of dedifferentiating upon tissue injury and which are the underlying mechanisms remain largely unclear.

Paneth cells are specialized secretory cells located at the very bottom of the crypt of Lieberkühn in the small intestine where they secrete antimicrobial peptides into the lumen^9^. Moreover, they provide essential physical support and secrete signals to ensure *Lgr5*^+^ stem cell function^10^. In the colon, where Paneth cells are not present, *cKit*^+^ Paneth-like cells (PLCs) play secretory and niche-like functional roles analogous to those of *bona fide* PCs in the small intestine^11,12^.

**Fig. 1 |:**
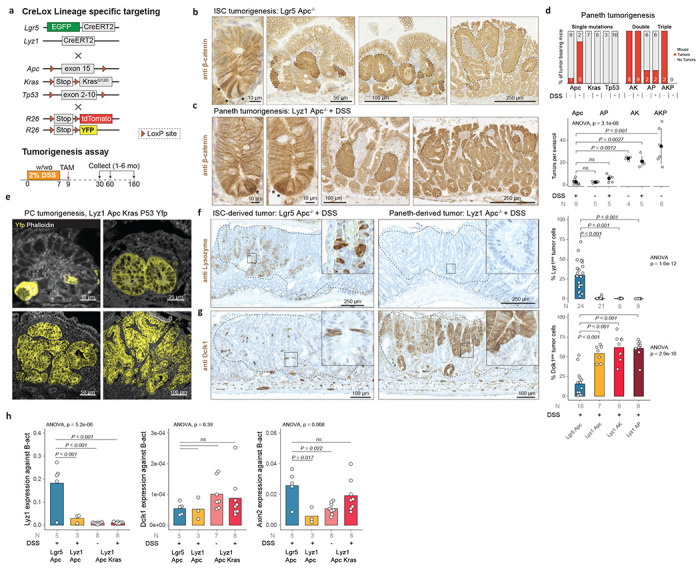
Paneth cells as the cell-of-origin of intestinal cancer. **a.** Cre-Lox strategy to target Apc, Kras, and Tp53 mutations in intestinal stem cells (Lgr5^+^ ISCs) and Paneth cells (Lyz1^+^ PCs). **b.** and **c.** β-catenin IHC analysis of intestinal tumors initiated from Lgr5^+^ ISCs (**b**) and PCs (**c**). The asterisks indicate Lgr5^+^ ISCs and PCs with enhanced cytoplasmic and nuclear β-catenin accumulation; tumor foci and adenomas are indicated by dashed lines. **d.** Tumor multiplicity was calculated according to tumor-bearing animals (top panel) and by tumor number per genotype (lower panel) in the presence/absence of DSS based on Swiss roll counts. Error bars denote standard deviations of the mean. P values denote one-way ANOVA and Tukey post-hoc tests for group comparisons. **e.** Lineage tracing analysis of Paneth cells (labelled by YFP) at different stages of tumor-initiation and progression. **f.** and **g.** Left panels: Lyz1 (**f**) and Dclk1 (**g**) IHC analysis Lgr5^+^ ISCs and PCs-derived adenomas. Right panels: quantification of number of Lyz1- and Dclk1-positive tumor cells. P values depict one-way ANOVA and Tukey post-hoc tests for group comparisons. **h.** *Lyz1, Dclk1*, and *Axin2* qPCR expression analysis across different adenoma genotypes. P values represent one-way ANOVA and Tukey post-hoc tests for group comparisons.

We first reported on the ability of Paneth cells to re-enter the cell cycle and de-differentiate upon irradiation and inflammation to acquire stem cell-like features and contribute to the tissue regenerative response^1,13,14^. Consequently, we questioned whether Paneth cells could be the origin of intestinal cancer in the context of inflammation. To this aim, we bred mice carrying Lox-alleles at the tumor suppressors and oncogenes most frequently mutated along the adenoma-to-carcinoma sequence, namely *Apc*^15^, *Kras*^16^, and *Tp53*^17^, each combined with a Cre specific for *Lgr5*^+^ ISCs (*Lgr5*^CreERT2-EGFP^)^18^ or for Paneth cells (*Lyz1*^CreERT2^)^19^. Following Cre activation by tamoxifen, DSS was administered through the drinking water to model inflammation (Fig. 1a). In the absence of DSS-induced inflammation, PC-specific single gene mutations did not give rise to intestinal tumors. In contrast, loss of *Apc* in *Lgr5*^+^-ISCs transformed crypts into β-catenin^hi^ foci that grew into adenomas 4-6 weeks after Cre induction (Fig. 1b). When single gene mutations were combined with DSS administration, *Apc* loss in Paneth cells resulted in increased nuclear and cytoplasmic β-catenin expression eventually leading to the formation of PC-derived adenomas (Fig.1c). Of note, Paneth-specific *Kras* or *Trp53* mutations did not result in tumor formation even in the presence of the inflammatory stimulus (Fig. 1d).

The compound loss of *Apc* and oncogenic activation of *Kras* in Paneth cells resulted in a striking increase in tumor multiplicity (6.1 fold) even in the absence of DSS (6.9 fold)(Fig. 1d). The combination of *Apc* and *Tp53* mutations in PCs also led to an increase in tumor multiplicity upon DSS administration (1.6 fold), though to a lesser extent when compared with the compound *Apc/Kras*-mutant genotype, possibly indicating a distinct mechanism underlying tumor onset in these mice. Indeed, phospho-histone H2A.X (Ser 139) IHC analysis confirmed an increase in DNA damage and chromosomal instability in the *Tp53*-mutant tumors (Suppl. Fig. 1a). Targeting all three genes in Paneth cells resulted in a very aggressive phenotype with high tumor multiplicity (10.1 fold) in the absence of the inflammatory stimulus (Fig. 1d). When compared to *Apc*-driven tumors originated in PCs, the histology of adenomas from mice in which two or three genes were targeted revealed a progressive increase in dysplasia and invasive morphology (Suppl. Fig. 1b).

To validate the Paneth cell origin of the observed intestinal tumors, we bred *Lyz1*^CreERT2^ mice with R26^LSL-tdTomato^ or R26^LSL-YFP^ reporters and traced their lineage upon tamoxifen-driven targeting of the *Apc*, *Kras*, and *Tp53* mutations. As shown in Figure 1e, this confirmed the Paneth cell origin of the corresponding tumors by capturing the process from microscopic lesions to adenoma formation.

Overall, these results demonstrate that Paneth cells can initiate intestinal adenomas upon genetic ablation of *Apc* in the context of inflammation. Activation of oncogenic *Kras* or loss of *Tp53* function rescues the need for an inflammatory stimulus and results in increased PC-derived tumor multiplicities and progression to malignant phenotype.

Next, we characterized lineage-specific markers in both the PC- and ISC-originated tumors by immunohistochemistry. Of note, while cells expressing the PC marker lysozyme (Lyz1) were notable in *Lgr5*-derived tumors (*Lgr5/Apc*: 30.0 % ± 18.5 positive tumor cells), they were nearly absent in adenomas that originated from Paneth cell mutation (*Lyz1*/*Apc*: 0.48 % ± 1.16)(Fig. 1f). The opposite was observed for Dclk1 (doublecortin like kinase 1), a Tuft^20^ and tumor stem cell marker^21,22^, that was more frequently detected among PC-derived adenomas (*Lyz1*/*Apc*: 54.1 % ± 10.5) when compared with *Lgr5*-derived tumors (*Lgr5*/*Apc*: 15.6 % ± 15.7) (Fig. 1g). Other lineage-specific markers for entero-endocrine (Chga), goblet (Muc2), and stem cells (Olfm4) showed variable levels but no difference among tumors with different cells-of-origin (Suppl. Fig. 2). The increased *Dclk1* expression in PC-derived tumors is of interest in view of its association with increased immune and stromal infiltration in colon cancer^23^.

To confirm these results at the transcriptional level, expression levels of the *Lyzl* and *Dclkl* genes were analyzed by RTqPCR (Fig. 1h). Indeed, *Lyz1* expression was lower in Paneth-derived tumors (*Lgr5*/*Apc* vs. *Lyz1*/*Apc*: log_2_FC = 2.64, P_val_ = 7.5e-4) when compared to *Lgr5*-derived tumors. *Dclk1* expression was very low and variable at the RNA level, and did not show significant differences across the groups. To assess the relative activation levels of the Wnt signaling pathway among the different tumor groups, we measured expression levels of *Axin2*, a well-established Wnt downstream target. *Axin2* expression was higher in *Lgr5*- compared to PC-derived tumors (*Lgr5/Apc* vs. *Lyz1*/*Apc*: log_2_FC = 2.12, P_val_ = 0.017)(Fig. 1h). Moreover, both *Kras* oncogenic activation and inflammation gradually increased *Axin2* levels in PC-derived tumors, in agreement with the previously reported synergism between *Apc* and *Kras* mutations in the activation of the Wnt pathway^24^. Thus, upon tumorigenesis, Paneth cells de-differentiate to a state that hampers differentiation toward Paneth cells, leading to specific patterns of tumor histology and gene expression distinct from that of *Lgr5*-derived tumors.

## Paneth cells dedifferentiate into revival stem cells upon enhanced Wnt signaling activation.

**Fig. 2 |:**
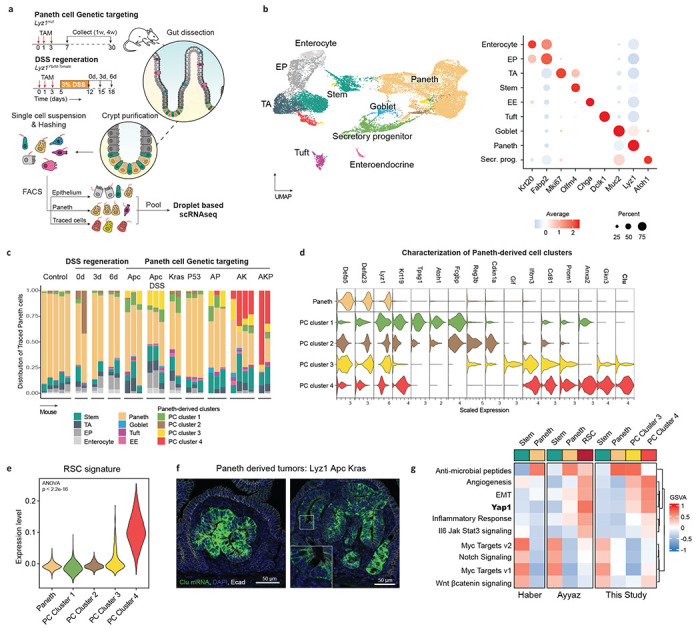
Paneth cells dedifferentiate into a revival stem cell identity. **a.** Schematics of the experimental approach. After genetic targeting of Paneth cells, intestinal crypts were extracted, and the isolated cells labeled with hashing antibodies and sorted according to three different sorting strategies: epithelium, PC-enriched and -traced cells. **b.** UMAP embedding of the different cell clusters/lineages (left panel), annotated according to the expression of canonical marker genes (right panel). **c.** Bar plot of the distribution of traced cells across the different mouse genotypes and experimental conditions. **d.** Violin plots representing marker genes of the newly identified Paneth-derived cell clusters (PC Cluster 1-4). **e.** Association analysis of the revival stem cell (RSC) signature with PC Cluster 1-4. **f.** RNA in situ hybridizations of the *Clu* gene in small tumors derived from Paneth cells upon compound targeting of *Apc* and *Kras* mutations. **g.** Gene sets variation analysis among the Haber et al.^55^, Ayyaz et al.^3^, and the present studies.

To elucidate the mechanisms which underlie the conversion of PCs into cells of origin of small intestinal tumors in the context of inflammation and/or of specific genetic hits, we combined scRNAseq analysis with lineage tracing. To this aim, we induced the *Apc*, *Kras*, and *Tp53* genetic mutations in the R26^LSL-tdTomato^/*Lyz1*^CreERT2^ (or R26^LSL-YFP^) reporter strains in the presence or absence of DSS (Fig. 2a). Subsequently, cells were harvested from the intestinal epithelium, purified by FACS, and transcriptionally profiled by scRNAseq (Methods; Suppl. Fig. 3). After preprocessing, we obtained the transcriptomes of 23231 epithelial cells from 32 mice, distributed over the different lineages of the intestinal epithelium (Fig. 2b). Close examination of the cells positive for the reporter genes (Methods) revealed novel clusters of PCs that arise upon DSS administration and the specific gene mutations, but were not observed among Paneth cells in homeostatic conditions (PC cluster 1-4, Fig. 2c).

To characterize the novel PC-derived states, we performed differential expression analysis and identified cluster-specific markers (Fig. 2d)(Suppl. Table 1). While PC Cluster 1 appeared at low frequency across different genotypes, PC Cluster 2 arises directly upon exposure to the inflammatory stimulus. Both PC Clusters 1 and 2 are characterized by increased expression of two markers of radio-resistant and secretory progenitors with self-renewal capacity during regeneration, namely *Krt19*^25^ and *Atoh1*^26^, while increased expression of *Reg3b*, known for its protective role in the development of colitis and ileitis^27^, and *Cdkn1a* (p21), a marker of terminal differentiation in the intestine^28^, was observed in Cluster 2 compared to 1. PC Cluster 3 became apparent in mice carrying *Apc* mutations (7.23% ± 5.77 of traced cells) alone and in combination with DSS treatment (16.40% ± 2.10 of traced cells), and in double and triple mutant (AP, AK, AKP) animals, though not in mice carrying single *Kras* or *Tp53* mutations. PC Cluster 3 showed increased expression of *Gif*(gastric intrinsic factor), *Cd81*, a tetraspanin family member known to mark the response to γ-irradiation and correlated with the expression of ISC- and proliferation genes^29^, and *Prom1* (prominin 1, also known as Cd133), a well-established colon cancer stem cell marker^30^.

PC Cluster 4 consisted of cells from mice where double (AK) and triple (AKP) mutations were targeted to Paneth cells (23.25% ± 11.23 and 52.26% ± 28.23 of traced cells, respectively). Increased expression of *Anxa2* (annexin 2), a functional marker of inflammatory response, and *Clu* (clusterin), previously shown to earmark revival stem cells (RSCs) upon γ-irradiation^3^, feature PC Cluster 4. Accordingly, evaluation of the RSC signature showed elevated expression among the PC clusters (Fig. 2e), and *in situ* hybridization analysis in PC-derived tumors from mice carrying mutations in both *Apc* and *Kras* (Fig. 2f) confirmed the increased *Clu* expression. Finally, pathway analysis revealed the similarities between the PC-derived Cluster 4 and RSCs, both earmarked by the activation of Yap1 signaling and specific inflammatory pathways (Fig. 2g). Of note, compared to RSCs, PC-derived and *Apc*/*Kras*-mutant cells from Cluster 4 showed increased levels of Tgf-β and Wnt signaling (Suppl. Fig. 4a).

Thus, upon genetic targeting or inflammatory stimulus, PCs escape their homeostatic identity and acquire distinct cellular features as shown by scRNAseq and FACS analysis (Suppl. Fig. 4b–c). Of note, DSS treatment led to a lower expression of *Lgr5* and *Ascl2* in stem cells, as well as a lower association of the CBC signature, confirming ours and others’ previous observations according to which resident stem cells lose their multipotency upon acute inflammation (Suppl. Fig. 4d)^1^.

Collectively, these findings demonstrate that PCs efficiently dedifferentiate upon genetic targeting or inflammatory stimulus leading to distinct cellular identities. During tumorigenesis driven by *Apc*/*Kras*, PCs share features with the Yap1-dependent revival stem cell identity, and further activate Tgf-β and Wnt signaling in their conversion to bona fide tumor cells.

## Transcriptomic comparison of Paneth- and *Lgr5*-derived tumors reveals a dichotomy in stem cell phenotypes.

**Fig. 3 |:**
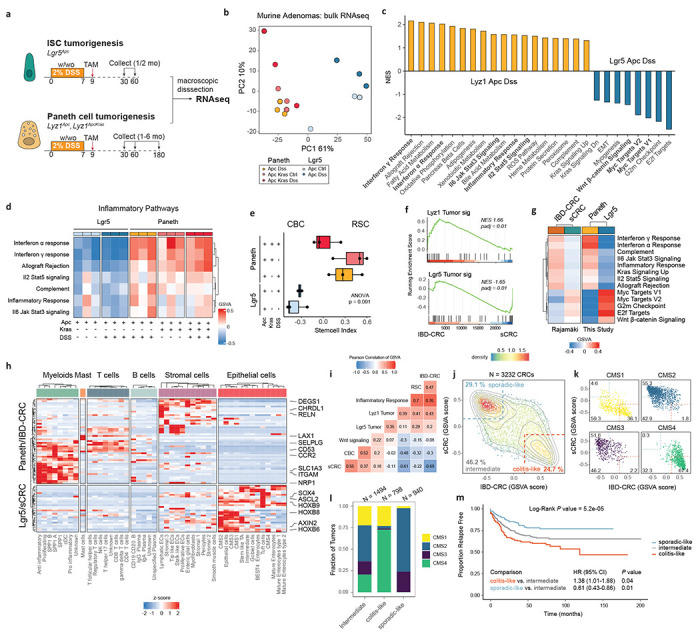
Paneth-derived adenomas have an inflammatory phenotype mimicking colitis-associated cancer **a.** Schematics of the experimental approach to compare PC- and Lgr5-derived adenomas. **b.** Principal Component Analysis plot showing that the cell-of-origin is the dominant discriminator of variance. **c.** Bar plot summarizing the gene set enrichment analysis between Paneth- (Lyz1 Apc DSS) and ISC- (Lgr5 Apc DSS) derived tumors. Pathways were filtered based on Pval < 0.05 and abs NES > 0.5. **d.** Subset of inflammatory pathways, visualized as heat map based on values from the gene set variation analysis. **e.** Box plots showing results of the stem cell index. P value depicts result of one-way ANOVA. **f.** Gene set enrichment analysis showing significant but opposite associations between the Lyz1 tumor signature and IBD-CRCs, and between the Lgr5 tumor signature and sCRC. **g.** Heatmap showing GSVA scores, averaged per tumor group, of pathways with similar patterns between the murine and human tumor groups. **h.** Heatmap highlighting the differentially expressed genes (log2FC >1.5, Padj < 0.01) shared between the Paneth/IBD-tumors and the Lgr5/sCRC-tumors. Values denote z-scores of average expression per cell type. **i-m.** Two distinct sporadic colon cancer identities become apparent upon analysis of a large cohort of CRC tumors (N = 3232 samples). **i.** Heatmap showing Pearson Correlations of the GSVA scores. **j-k.** Scatter plot showing the two distinct clusters of sporadic- and colit6is-like in all (**j**) colon cancers and (**k**) per molecular subtype. Grey lines indicate contours lines, dashed lines show thresholds to classify tumors in colitis-like, sporadic-like and intermediate groups. **I.** Stacked bar plot analysis showing the distribution of consensus molecular subtypes (CMS1 to 4) across the colitis-like and sporadic-like colon cancers. **m.** Kaplan-Meier survival analysis for relapse free survival. Pvalues denote result of log-rank test and cox regression models for univeriate analyses. Hazard ratios (HR) and confidence intervals (CI) are displayed for pairwise comparisons.

To investigate the consequences of the cell-of-origin identity on the transcriptional profile of the resulting intestinal tumors, we performed bulk RNA sequencing of macroscopically dissected lesions originated from ISCs and PCs (Fig. 3a). Principal component analysis revealed that the major variance component (61%) was attributed to differences in the cell-of-origin, while the impact of genotype or inflammatory stimulus became notable in the second component of variation (10%; Fig. 3b). Differential expression analysis between tumors derived from PCs and ISCs in the same genetic and inflammatory context (*Apc* and DSS) revealed tumor signatures specific for each cell-of-origin (Suppl. Fig. 5a, and Suppl. Table 2).

Gene set enrichment analysis (GSEA) indicated that tumors derived from ISCs were characterized by high levels of Myc and Wnt signaling, while PC-derived adenomas showed higher levels of inflammatory pathways (Fig. 3c). Of note, the inflammatory characteristics of PC-derived tumors were observed also in mice where *Apc* and *Kras* mutations were targeted to Paneth cells in the absence of DSS-driven inflammation (Fig. 3d), indicating that specific mutant genotypes and the type of cell-of-origin can trigger tumor initiation by mimicking the inflammatory context otherwise brought about by DSS.

Next, we employed the ISC index^4^ (intestinal stem cell index; Methods) to predict the relative proportions of RSC and CBC stem cells in the intestinal tumors. In agreement with the scRNAseq analysis, PC-derived tumors were RSC-enriched whereas the ISC-derived tumors consisted mainly of CBCs (Fig. 3e). Notably, the highest RSC contribution was observed in tumors originated from *Apc*/*Kras*-mutant PCs in the absence of inflammation, when compared with the equivalent genotype upon DSS administration.

Together, these results indicate that the cell-of-origin embodies the major source of inter-tumor variability, and that Paneth-derived tumors exhibit an inflammatory RSC-like profile while ISC-derived tumors encompass a more CBC-like profile, earmarked by the activation of Wnt signaling and Myc target genes.

## The transcriptional profile of Paneth-derived tumors mimics colitis-associated colorectal cancer.

In view of the marked differences in the transcriptional profiles between mouse intestinal tumors with distinct cells of origin, we questioned whether similar differences distinguished human sporadic colon cancers from those that arose in the context of IBD. To this aim, RNAseq profiles of human microsatellite stable sporadic colorectal cancers (sCRC, N = 38) and from patients suffering from inflammatory bowel disease (IBD-CRC, N = 14) were interrogated^31^. GSEA of the most differentially expressed genes (log_2_FC > 5, P_adj_ < 0.01) from the mouse tumors (*Lyz*T signature, N = 27 genes; *Lgr5*T signature, N = 40 genes) revealed a significant association between the *Lyz*T signature and IBD-CRC (NES 1.67, P_adj_ 6.2e-3), while the *Lgr5*T profile was significantly associated with sCRC (NES −1.62, P_adj_ 0.013)(Fig. 3f, Suppl. Fig. 5b, Suppl. Table 3). Evaluation of the hallmarks from the molecular signature database^32^ revealed gene sets common to PC-derived tumors and IBD-related CRCs (Interferon α/γ, inflammatory response, Il6/Il2 signaling, Kras, Complement, Allograft rejection), and to *Lgr5*-derived tumors and sporadic CRCs (Myc targets, G2m Checkpoint, E2F targets and Wnt β-catenin signaling)(Fig. 3g, Suppl. Fig. 5c).

We then compared the differentially expressed genes from PC- versus *Lgr5*-derived mouse tumors and human IBD-CRC versus sCRC (Paneth/IBD-CRC, N = 49 genes, Lgr5/sCRC: N = 27 genes) and visualized their expression across different cell types based on a large scRNAseq CRC study^33^ (Fig. 3h). Of note, the markers shared between PC-derived tumors and IBD-CRC were dominantly expressed in myofibroblasts (e.g. *ITGAM*, *SLC1A3*), T cells (e.g. *SELPLG*, *LAX1*), and stromal cells (e.g. *CHRDL1*, *RELN*). In contrast, *Lgr5*-derived tumors/sCRC markers were mostly observed in epithelial cells (e.g. *HOXB6, HOXB8, HOXB9, AXIN2, ASCL2*), indicating the difference in stromal composition among these tumors (Fig. 3h). Comparison of a set of gene signatures (Methods, Suppl. Table 4) in a large colorectal cancer cohort^34^ confirmed the presence of two distinct identities (Fig. 3i,j): a colitis-like identity enriched with RSCs and prevalent in CMS4 (67%) and CMS1 (36%) tumors, and a sporadic-like identity enriched with CBCs and common in CMS2 (55%) and CMS3 tumors (52%)(Fig. 3k,l, Suppl. Fig 5d). Survival analysis revealed significant differences in relapse free survival between the sporadic- and colitis-like CRC groups (Pvalue = 5.2e-05)(Fig. 3m). Thus, transcriptional signatures derived from small intestinal mouse tumors originated from Paneth cells significantly overlap with those from human colon cancer that arose in the context of IBD, possibly revealing a common cell-of-origin in secretory lineages.

## Western-style diet triggers an inflammatory response leading to dedifferentiation of Paneth cells.

The representation of the colitis-like identity observed among sporadic colon cancers (24.7%; Fig. 3j) vastly exceeds the expected proportion of colon cancers arising in patients with an IBD history (1-2%)^35^. One possible explanation for this apparent discrepancy may come from a recent report by J.C. ad L.H.A. where a purified mouse diet that mimics the western-style dietary habits associated with increased risk of colon cancer (NWD1^36^), was shown to induce a low degree of chronic intestinal inflammation and other mechanisms that define pathogenesis of human IBD^37^. Therefore, we hypothesized that etiologic drivers for colon cancer other than IBD, including widespread western-style dietary habits, may underlie dedifferentiation and tumor onset mechanisms similar to those observed upon acute DSS-driven inflammation.

**Fig. 4 |:**
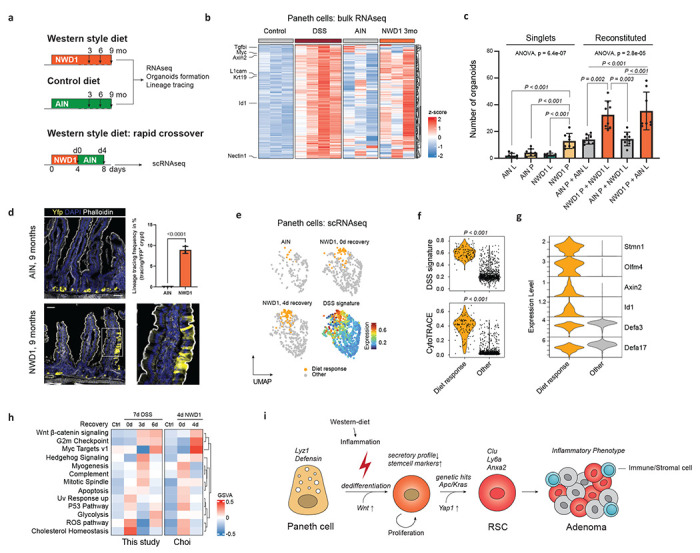
Western-style diet triggers an inflammatory response leading to dedifferentiation of Paneth cells. **a.** Schematics of the experimental approach to investigate the consequences of short- and long-term exposure to western-style diet (NWD1) vs. control (AIN76A) diets. **b.** Heatmap showing z-scored DSS signature (DSS vs. Control; P_adj_ < 0.05, Log_2_FC > 0.25) in Paneth cells exposed to DSS or NWD1. **c.** Organoid multiplicities derived either from single ISCs and PCs and reconstituted doublets (L: Lgr5^+^ ISCs, P: Paneth cells). Pooled data from N = 4 independent experiments. P values were calculated using one-way ANOVA and Tukey tests for group comparisons. Error bars depict standard deviations from the mean. **d.** Representative image of lineage tracings from a NWD1-fed Lyz1-Yfp mouse. Scale bar: 50 μm. P value depicts result of the student t-test and error bar represents the standard deviation. Data from N = 3 mice. **e.** UMAP showing Paneth cells from AIN76A- and NWD1-fed mice (N = 3 mice per condition). The DSS signature portrayed on UMAP embedding, highlights a subcluster of Paneth cells responsive to the NWD1 diet. **f.** Violin plots showing different levels of the DSS signature (top) and CytoTRACE score (bottom) between the PCs responsive to the NWD1 diet and other Paneth cells. P values depict significance values of Wilcoxon test. **g.** Violin plots representing marker genes of PCs responsive to the NWD1 diet, showing co-expression of stem and secretory markers. **h.** Heatmap visualization of gene set variation analysis, indicating pathways that are activated in Paneth cells after exposure to DSS or NWD1. **i.** Model: Paneth cells react to dietary and inflammatory cues by dedifferentiation to aid epithelial regeneration. During this process, Paneth cells become potential cells-of-origin of cancer, leading to an alternative bottom-up route to intestinal tumorigenesis.

To clarify this potentially relevant observation, we first fed C57BL6/J mice for 3 months with western-style (NWD1) and control (AIN76A) diets and compared the transcriptional response of Paneth cells with that obtained upon DSS administration (Fig. 4a). We examined genes upregulated upon inflammatory stimuli (DSS signature; see Methods), which showed variable but overall increased levels in NWD1-fed mice when compared to those on the control diet (Fig. 4b). Indeed, GSEA confirmed that the DSS signature was associated significantly with Paneth cells exposed to NWD1 (NES 2.99, P_adj_ < 0.001), indicating that western-style dietary habits trigger an inflammatory-like response in Paneth cells (Suppl. Fig. 6a). At the gene ontology level, western-style diet activated signaling pathways related to cell cycle (G2M Checkpoint) and proliferation (mitotic spindle, Myc targets; Suppl. Fig. 6b), suggesting that Paneth cells re-enter the cell cycle upon long-term exposure to western-style diet, similar to what observed upon DSS-driven inflammation^1^.

In view of the previously reported acquisition of stem-like features by PCs upon inflammation^1^, we next tested the organoid formation capacity of Paneth and *Lgr5*^+^ cells from mice fed with the western-style and control diets. As depicted in Fig. 4c, single Paneth cells from NWD1-fed mice formed organoids more efficiently compared with PCs from AIN76A-fed mice and with *Lgr5*^+^ ISCs from both groups of animals. As the results obtained with single PCs may be confounded by the presence of doublets even after their depletion by FACS^14^, we performed organoid reconstitution assay (ORA)^14,38^ by co-incubating Paneth and *Lgr5*^+^ cells from the AIN76A- and NWD1-fed mice in all 4 combinations. Paneth cells from NWD1-fed animals significantly improved organoid formation independently of their reconstitution with *Lgr5*^+^ cells from NWD1- or AIN76A-fed mice (Fig. 4c). Furthermore, lineage tracing analysis of Paneth cells in the R26^LSL-YFP^*Lyz1*^CreERT2^ reporter strain revealed extended YFP-labeled ribbons in NWD1-fed mice thus confirming their dedifferentiation and acquisition of stem-like features induced by the western-style dietary cues (Fig. 4d).

To zoom in on the primary transcriptional response of Paneth cells to NWD1, we took advantage of the scRNAseq data generated by J.C., L.H.C., and collaborators immediately upon exposure to NWD1^37^ (Fig. 4a). Within 4 days of switching mice to the NWD1 diet, a subset of Paneth cells became apparent whose transcriptional profile strongly associated with the DSS signature (labeled as “Diet response cells” in Fig. 4e). Mirroring our previous observations obtained immediately upon DSS inflammatory stimulus, these NWD1-responsive cells increased their transcriptomic diversity as measured by CytoTRACE^39^ (Fig. 4f, Methods). Already after the short exposure to NWD1, the diet-responsive cells acquired stem cell markers while retaining some secretory features (Fig. 4g). Comparative pathway analysis between the transcriptional response of Paneth cells to DSS and NWD1 revealed similar upregulation of Wnt, Myc, Hedgehog, and G2M checkpoint signaling pathways (Fig. 4h).

Collectively, our results reveal an alternative bottom-up route to intestinal tumorigenesis originating from Paneth cells in the murine small intestine and presumably from secretory precursors (Paneth-like cells)^11,14^ in the human colon, likely triggered by inflammatory cues either directly as in IBD, or through western-style dietary factors. Along this dedifferentiation process, Paneth cells progress through a revival stem-like state driven by the combined activation of Yap1, Tgf-β, and Wnt signaling. Compared to those arising from ISCs, PC-derived tumors are earmarked by distinct inflammatory features reminiscent of colitis associated cancer (Fig. 4i). Accordingly, the increased expression of Dclk1 in the murine PC-derived tumors is reminiscent of the association of this Tuft and cancer stem cell marker with human colon cancers earmarked by increased immune and stromal infiltration and poor prognosis (CMS4)^23^. As such, dedifferentiation of committed (secretory) lineages may lie upstream of DCLK1 activation in colon cancer and characterize a subset of patients eligible for immune therapy^40^.

*Lgr5*^+^ stem cells have been established as the origin of intestinal tumors^5^. However, whether the same holds true in the context of inflammation has been challenged by their loss of lineage tracing capacity and multipotency upon a broad spectrum of tissue injuries ranging from DSS-driven inflammation^1^ to *γ*-irradiation^25,41^, and western-style diet^42^. Our results show that Paneth cell de-differentiation and tumor onset are triggered by inflammation and loss of *Apc* function. Compound PC-specific *Apc*- and *Kras* mutations elicit tumorigenesis even in the absence of the inflammatory stimuli thus revealing the central role played by enhanced Wnt signaling and proliferation in underlying tumor onset as a consequence of the regenerative response. As such, the increased cancer risk in IBD patients may reflect on a ‘trade-off’ effect where the chronic response to inflammation induces continuous de-differentiation of committed lineages into stem-like cells to support tissue regeneration, thus resulting in the enlargement of the pool of potential targets for tumor onset. Relative to the debate on the relative contribution of extrinsic risk factors versus the rate of stem cell division to cancer development^43,44^, it appears that colon cancer etiologic factors such as inflammation and poor dietary habits are likely to result in quantitative and qualitative alterations of the stem cell niche which ultimately predispose to neoplastic transformation. The resulting subset of tumors follow distinct evolutionary paths when compared to Wnt/Myc-driven *Lgr5*-derived tumorigenesis, and are characterized by an inflammatory tumor phenotype more prone to infiltration from the tumor micro-environment.

## Methods

### Mice

The following inducible Cre strains encompassing were employed: *Lgr5*^CreERT2-EGFP^ (#008875, Jackson Lab^18^) and p*Lys*^CreERT2^ (kind gift from the Clevers Lab^19^). For lineage tracing experiments, mice were crossed with R26^LSL-YFP^ mice (#006148, Jackson Laboratories) or R26^LSL-tdTomato^ mice (#007908, Jackson Laboratories). To target specific mutations in PCs, the above strains were further crossed with *Apc*^15lox^ (#029275, Jackson Laboratories)^15^, *Kras*^LSL-G12D^ (#008179, Jackson Laboratories)^16^, and *Tp53*^flox^ (#008462, Jackson Laboratories)^17^. C-recombinase was activated by intraperitoneal injections of Tamoxifen (1 mg dissolved in 100% EtOH and subsequently in sunflower oil; #T5648 and #S5007, Sigma) once or three times in 4 days. To induce acute intestinal inflammation, mice were administered 2-3% dextran sodium sulfate (DSS) in their drinking water for 7 days (#0216011050, MP Biomedicals). For diet experiments, mice were fed with AIN76A or NWD1 diet^42^ and collected at various time points (4/8 days, 3/6/9 months). For all experiments, mice were randomly assigned to experimental groups after matching for gender, age of 8-12 weeks, and genotype. All protocols involving animals were approved by the Dutch Animal Experimental Committee and in accordance with the Code of Practice for Animal Experiments in Cancer Research established by the Netherlands Inspectorate for Health Protections, Commodities and Veterinary Public Health. Animals were bred and maintained in the Erasmus MC animal facility (EDC) under conventional specific pathogen-free conditions.

### Lineage tracing

*Lys*_CreERT/R26LSL-YFP_ mice were injected three times with Tamoxifen (1 mg, i.p.) on consecutive days. One week after the last Tamoxifen injection, intestinal tissues were harvested for lineage tracing analysis. To this aim, tissue samples were first dissected and washed with PBS, and then fixed for 2 hours at 25°C with 4% buffered formaldehyde solution (Klinipath). Tissues were cryo-protected in 30% sucrose (Sigma) overnight at 4°C, embedded in OCT (KP cryocompound, Klinipath), frozen on dry ice, and sectioned at - 20°C. Tissues were cut in 4–8 μm thick sections. These sections were incubated in PBS containing Alexa 568 Phalloidin (1:100; Invitrogen) or Alexa 633 Phalloidin (1:100; Invitrogen), and DAPI (Sigma) for 30 minutes at 25°C and washed in PBS-T. Tissues were mounted in Vectashield Mounting Medium (Vector Labs) and imaged with a LSM700 confocal microscope (Zeiss). Images were processed with ImageJ. Lineage tracing frequency was quantified by dividing the number of intestinal crypt/villus axes containing Yfp^+^ ribbons (encompassing at least 5 Yfp^+^ labelled cells) by the total number of counted Yfp labelled crypts. Lineage tracing analysis was performed on 3 mice for each diet type; in each mice at least 60 YFP labelled crypts were analyzed.

### Immunohistochemistry

Tissues were fixed in 4% PFA overnight at 4°C and embedded in paraffin. The 4 μm sections were dewaxed with Xylene and hydrated in consecutive rounds of 70% and 100% EtOH. Antigen retrieval was performed in the 2100 Retriever pressure cooker (BioVendor) at pH 9 with Tris-EDTA buffer. After a 10 minutes incubation at RT with 3% hydrogen peroxidase, tissues were blocked with 5% skim milk powder (Milipore) in PBS-Tween. The following primary antibodies were employed by overnight incubation at 4°C: β-catenin, #610154 (BD Biosciences); μ-H2AX, #9718 (Cell Signaling); Gfp, #A-11122 (ThermoFisher); Olfm4, #D6Y5A (Cell Signaling); Lyz1, #A0099 (Dako); Dclk1, #ab37994 (Abcam); Muc2, #sc-15334 (Santa Cruz); Chga, #NB120-15160 (Novus Bio); Ecad, #610182 (BD Biosciences). Slides were washed twice with PBS-Tween and incubated for 30 minutes at room temperature with the Rabbit/Mouse EnVision kits (K4001/K4007, Dako). Slides were counterstained with hematoxylin and, dehydrated with subsequent 70% and 100% EtOH and mounted with Pertex (00811, Histolab). Whole slides were scanned with the Nanozoomer (Hamamatsu) and analyzed with NDP viewer v2 (Hamamatsu).

### *In situ* hybridization

In situ hybridization was performed by RNAscope^™^ Multiplex Fluorescent V2 Assay (ACDBio) technology in combination with the RNA-Protein Co-Detection kit (ACDBio), according to the manufacturer’s instructions. In brief, 4 μm paraffin embedded sections were dehydrated, blocked for 10 minutes at room teperature with H_2_O_2_, and processed with 1X Antigen Retrieval co-detection buffer for 15 minutes at 99°C. Sections were incubated with Ecad (1:250, #610182, BD Biosciences) overnight at 4°C. Hybridization was performed with a probe targeting mouse *Clu* (Cat#427891, ADCBio), and the probe signal was developed with Vivid dye 520 nm (1:1500). Sections were counterstained by DAPI and anti-mouse Alexa 633 (1:250, Invitrogen) and mounted with ProLong^™^ Gold Mountant (ThermoFisher). Samples were imaged on the Stellaris 5 Confocal Microscope (Leica) and images were processed with ImageJ.

### Image analysis

Images were scanned by Nanozoomer and imported in QuPath^45^ as IHC images. Within intestinal tumors, regions of interest (ROI) were randomly assigned and consisted of 300-600 cells. Next, the ROIs were processed with positive cell selection plugin using default settings based on Cell DAB OD mean. The percentage of positive cells per ROI was exported and visualized in R, where statistical tests were performed.

### Quantitative real-time PCR

RNA was converted into cDNA using the High Capacity RNA-to-cDNA kit (Applied Biosystems). Samples were run in duplicates on the 7500 Real Time system (Applied Biosystems). Quantitative PCR was performed using the TaqMan assay (Applied Biosystems) according to instructions of the manufacturer. Samples were normalized to the beta-actin (*Actb*) house-keeping gene. The gene-specific Taqman probes were: Mm02619580_g1 (*Actb*), Mm00443610_m1 (*Axin2*), Mm00657323 (*Lyz1*), Mm01545303 (*Dclk1*). Data were processed and visualized in R and statistical tests were employed to assess significance with one-way ANOVA and Tukey post hoc test.

### Isolation of mouse intestinal crypts and cell dissociation

Mouse small intestines were flushed with cold PBS, removed from fat and scraped with a glass slide to remove intestinal villi. Resected sample were then sectioned into 5-10 mm segments and washed with cold PBS before incubation for 30 minutes at 4°C in cold PBS supplemented with 6 mM EDTA. After an additional PBS wash, crypts were detached from the muscle layer by 4 rounds of harsh pipetting with cold PBS. Crypts were treated for 10 minutes at room temperature with DNAse (2000 U/ml-1, Thermo Scientific) in Advanced DMEM/F-12 medium and passed through a 70 μm strainer. Purified crypts were dissociated with 1 mL of pre-warmed TryplE and DNAse for 3 minutes at 37°C. Single cells were pelleted in 10 mL ADF, resuspended in 3 mL 5% FCS HBSS and manually counted.

### Fluorescence activated cell sorting

Cell suspensions were blocked with TruStain Fc blocking reagent (Biolegend) for 10 minutes at 4°C. After washing with 5% FCS HBSS, cells were stained for 30 minutes at 4°C in 100 μl with Lin antibodies (CD31-BV421 #563356, CD45-BV421 #563890, TER119-BV421 #563998, BD Biosciences), CD24-APC (#1109070, Sony Biotechnology), and cKit-PE (#105808, Biolegend). For scRNAseq experiments, the antibody mix was supplemented with 2 μl Hashing Antibody (Biolegend TotalSeq^™^ Hashtag antibodies: #155831, #155833, #155837, #155843, #155849) to label the cells with oligo barcodes. After 3 washes with 3.5 mL 5% FCS HBSS, cells were filtered with a 40 μm strainer and resuspended in 1 mL 5% FCS HBSS with 1 μg/mL DAPI (D9542, Sigma-Aldrich). FACS analysis and sorting were performed using a FACSAriaIII (BD Biosciences). DAPI and BV421-conjugated antibodies were detected using a 405nm laser and 450/40 BP filter. GFP and BB515-conjugated antibodies were detected using a 488nm laser and 502LP + 530/30 BP filters. tdTomato and PE-conjugated antibodies were detected using a 561nm laser and 582/15 BP filter. APC-conjugated antibodies were detected using a 633nm laser and 660/20 BP filter. Samples were sorted for epithelial (Lin^neg^), Paneth-enriched (SSC^hi^CD24^hi^cKit^hi^), and traced cells (Yfp^hi^/td-Tomato^hi^) according to previously established gates^1,13,14^. To optimize the sorting for single cell experiments, the initial single cell gate was followed by a more stringent, population specific single cell gate^13^. FACS strategy was visualized with FlowJo V10.

### Organoid Reconstitution Assay

Whole crypts were extracted from the small intestine of *Lgr5*-EGFP mice fed for 3 month (from weaning) with either AIN76A or NWD1 diets^42^. After cellular dissociation and FACS, single *Lgr5*^+^ and Paneth cells were sorted in separate Eppendorf LoBind Tubes. Reconstitution of *Lgr5*-EGFP^hi^ stem cells with PCs (SSC^hi^CD24^hi^cKit^hi^) was performed by co-pelleting sorted cells at 300 g for 5 minutes in Eppendorf LoBind Tubes, and by incubating them for 15 minutes at 25°C as previously described^14,38^. The cells were then resuspended in 30 μL Matrigel (Corning), and cultured in 96 Well Flat Bottom dishes (Corning). Additionally, Paneth and Lgr5 positive cells were plated singularly where indicated and cultured in standard ENR medium^41^ supplemented with 10 μM Y-27632 (Sigma) and 1 mM Jagged-1 (AnaSpec). Cells were plated in triplicates and the generated organoids where counted 9 days after plating. Experiments were repeated at 3 independent moments.

### Droplet-based scRNAseq

Sorted single cells were mixed in equal sample proportions to a pool of 25-50k cells in 5% FCS HBSS to a final concentration of ±1000 cells/μl. The cell pool was recounted manually with Trypan blue and between 10-15k cells were loaded into a 10X Genomics Chip (Next GEM Chip G). The Gel Bead-In EMulsions were generated with the Chromium Controller (10X Genomics) using Single Cell 3’ v3 chemistry with FeatureBarcoding. After generation of cDNA and quality check with Bioanalyzer (Agilent), libraries were sequenced with the Novaseq 6000 (Illumina) till a depth of 20-40k reads/cell. Raw data was processed with CellRanger count (cellranger-7.0.0)^46^ and aligned to the mm10 mouse reference genome with the addition of the transgenes Yfp and Td-tomato.

### Analysis of scRNAseq data

For each single cell run, filtered gene-cell matrices were imported in R using Seurat (v 4.1.1)^47^. After a centered log ratio transformation (CLR) of the hashtag oligo counts, cells were assigned back to their mouse of origin using HTODemux (positive quantile = 0.99). Cells assigned as doublets or negative for any of the barcodes were filtered from the data. Cells from mice with different gender but same genotype and experimental group were further separated based on the expression *Xist*. After preprocessing, the five runs were merged into a single Seurat object. To remove putative doublets from the sorting, doublets were simulated and called by doubletfinder_v3 (nExp = 0.01)^48^. After removal of doublets, low quality cells were removed by filters based on the percentage of mitochondrial genes (> 10%), on nFeature_RNA (< 200), and nCount_RNA (< 500). Next, data were integrated with the single cell transform (SCT) pipeline using reciprocal PCA dimension reduction to find integration anchors. Integrated data was further processed by dimension reduction with Uniform Manifold Approximation and Projection (UMAP) on the first 30 principal components. Cells were clustered using shared nearest neighbor (SNN) modularity optimization (resolution = 0.6) and annotated according to canonical marker genes. Traced cells were defined as cell with a non-zero value for either td-Tomato or Yfp. Signatures were evaluated using the Addmodulescore function and visualized in experimental groups or cell clusters using Violin Plots. Pathway level evaluation was done using the GSVA package^49^ on summarized expression profiles using Average Expression by cell cluster.

### Bulk RNAseq of intestinal tumors

Intestinal tumors were macro-dissected from intestinal tissues and cut into 1-2 mm small fragments which were then re-suspended in 500 μl TRIzol (#15596026, Ambion) for RNA isolation. RNA quality was first evaluated by Nanodrop ND-1000 (ThermoFisher) and further purified with the TURBO DNA-free Kit (Invitrogen). Samples were sequenced with a read length of 150 bp (PE150) using the DNA nanoball (DNB-seq) protocol till a depth of 25M reads/sample (BGI, Hong Kong). Adapter trimming and sequencing QC was performed with SOAPnuke software^50^ .

### Analysis of mouse tumors: bulk RNAseq

The FASTQ files were aligned with STAR (v2.7.9a) to the mm10 mouse reference genome with Ensemble gene annotations^51^. After alignment, Sambamba (0.8.0) was applied to mark duplicates and perform flagstat quality checks^52^. Next, Subread (2.0.3) was used to count the primary strand-specific alignment^53^. Downstream analysis was performed in R using the DeSeq2 package (v 1.34.0)^54^. In brief, counts were log2 normalized and used for principal component analysis on the top 500 variable expressed genes. Differential expression analysis was done following the DeSeq pipeline with Negative Binomial GLM fitting and Wald statistics and genes were filtered using log_2_FC >1.5, P_adj_ < 0.01. Gene set enrichment analysis was done with the fgsea package (v 1.20.0) using the HallMarks gene modules from the molecular signature database^32^. Gene set variation analysis was performed using the GSVA package (v 1.42.0)^49^. The relative proportion of RSC/CBC stem cell types was computed with the ISC index using default settings^4^.

### Analysis of Paneth cells upon exposure to inflammation and western-style diet: bulk RNAseq

The FASTQ files were removed from TrueSeq adapters using Trimmomatic (v0.36). STAR was used to align the reads to the mm10 reference genome using Gencode annotation release M15 (GRCm38.p5)^51^. Mapping quality was assessed with Sambamba Flagstat statistics. Count files were generated using FeatureCounts (subread) and further processed in R using the DeSeq2 package. Differential expression analysis were performed between the groups ‘NWD1 diet’ (n = 3) vs. ‘AIN76 diet’ (n = 3) with the Wald-test. A heat map was produced using z-score scaled data and visualized with the ComplexHeatmap package (v2.12.1).

### Analysis of IBD-CRC and sCRC RNAseq cohort

RNA-seq material was previously described in Rajamäki et al.^31^ In brief, 64 CRCs entered HiSeq LncRNA-Seq library preparation and paired-end sequencing using Illumina HiSeqXTen. Salmon (version 0.12.0; quant mode with ValidateMappings) was used to map raw sequences onto the human transcriptome (Ensembl release 79). Gene-level quantification was done with DESeq2 (vl.18.1) followed by a variance stabilizing transformation and Limma (v3.34.9) correction of sequencing batch effects^54^. Only microsatellite stable (MSS) tumors were included in the analyses. Differential gene expression was calculated as ANOVA between 38 MSS sCRCs and 14 MSS IBD-CRCs. GSVA (v1.42.0)^49^ analyses were ran using default options (Gaussian kernel) and predefined gene sets from the molecular signature database^32^.

### Analysis of human CRC bulk RNAseq cohort

Log2 normalized expression of the meta cohort described in Guinney et al.^34^ was obtained from the R2 Genomics Analysis and Visualization Platform and subsequently processed in R. Gene set variation analysis was performed with the GSVA package using the Gaussian kernel^49^. Subsequently, correlations of the gene signatures (Suppl. Table 4) were visualized with ggcorr using Pearson Correlation. Next, samples were classified as colitis-like, sporadic-like or as intermediate based on their GSVA scores for the IBD-CRC and sCRC signatures. Using this classification, samples were visualized with their annotated Consensus Molecular Subtype (CMS). Survival analysis was performed with the survival and survminer packages in R. Multivariate analysis was performed with the log-rank test and pairwise comparisons were fitted with a Cox proportional hazards regression model.

### Analysis of human CRC scRNAseq cohort

Filtered count matrices were retrieved from the gene expression omnibus (KUL cohort: GSE144735; SMC cohort: GSE132465)^33^ and processed in R with Seurat. After normalization and scaling of the Seurat object, gene expression was summarized per cell type using AverageExpression based on the overlapping differentially expressed genes of murine (Paneth vs. *Lgr5*) and human (IBD-CRC vs. sCRC) tumor groups. Last, the average expression was z-scored and visualized as a heat map using the ComplexHeatmap package.

### Analysis of mouse intestinal scRNAseq: homeostasis and γ-irradiation

Count matrices from Haber et al.^55^ and Ayyaz et al.^3^ were downloaded from GEO using GSE92332 and GSE117783, respectively. After preprocessing using the Seurat workflow, Seurat objects were merged and gene expression was averaged by cell type and study using AverageExpression. Next, gene set variation analysis (GSVA)^49^ was computed using the Gaussian kernel and the output was visualized in a heat map using the ComplexHeatmap package.

### Analysis of mouse intestinal scRNAseq: AIN76A and NWD1 diets

The intestinal epithelia of mice fed with control (AIN76A) and western-style diets (4 days NWD1, 4 days NWD1 + 4 days AIN recovery) were collected and sent for 10X Genomics scRNAseq analysis (N = 3 mice per group)^37^. After preprocessing with Seurat and batch correction using the integrated assay workflow, the Seurat clusters were annotated according to canonical markers. CytoTRACE^39^ was computed on the whole data set using default settings. Next, Paneth cells (N = 991 cells) were taken from the data set, subclustered, and visualized by UMAP with a split by the dietary condition. The DSS signature was computed using AddModuleScore and projected on the UMAP embedding. The Paneth subcluster that appeared in the NWD1 conditions and associated with the DSS signature was relabeled as ‘Diet response’. Next, marker genes were visualized using ViolinPlot based on the separation of diet response PCs and other PCs. Last, Paneth cells from the diet data set were merged with the Paneth cells from the DSS data set. Average expression was computed based on experimental condition, and GSVA analysis (Gaussian kernel) was performed using the predefined gene sets from the Molecular Signature Database (Hallmark set)^32,49^. A filter was applied to select pathways that were both upregulated in DSS and NWD1 condition (ΔGSVA = 0.2). The output was visualized as heat map using the ComplexHeatmap package.

### Data availability

All data relevant to this study are made available. Transcriptomic sequencing data has been deposited in the Gene Expression Omnibus (GEO), and is available using the following identifiers: GSE221819 (bulk RNA sequencing of murine tumors); GSE221820 (single cell RNA sequencing of genetically targeted Paneth cells); GSE221818 (bulk RNA sequencing of sorted Paneth cells treated with control and western-style diet). Additional data sets referenced in this study are publicly available in the Synapse and GEO repositories. Previously published expression profiling studies were employed relative to the mouse small intestine in homeostasis (Haber et al.^55^, GSE92332), upon irradiation (Ayyaz et al.^3^, GSE117783), upon inflammation (Schmitt et al.^1^, GSE117216) and upon feeding with western-style diet (Choi et al.^37^, GSE188577). Human colorectal cancer Bulk RNAseq and scRNAseq studies were retrieved from Guinney et al.^34^ (syn2623706), Rajamäki et al.^31^ and Lee et al.^33^ (KUL cohort: GSE144735; SMC cohort: GSE132465), respectively.

## Supplementary Material

1
