# Supplementary material for: Paneth cells as the origin of intestinal cancer in the context of inflammation
Source: Res Sq. 2023 Jan 19:rs.3.rs-2458794. Preprint. [Version 1] doi: 10.21203/rs.3.rs-2458794/v1 (PMC9882659; doi:10.21203/rs.3.rs-2458794/v1)
Supplement: 1 [file NIHPPrs2458794v1-supplement-1.pdf]

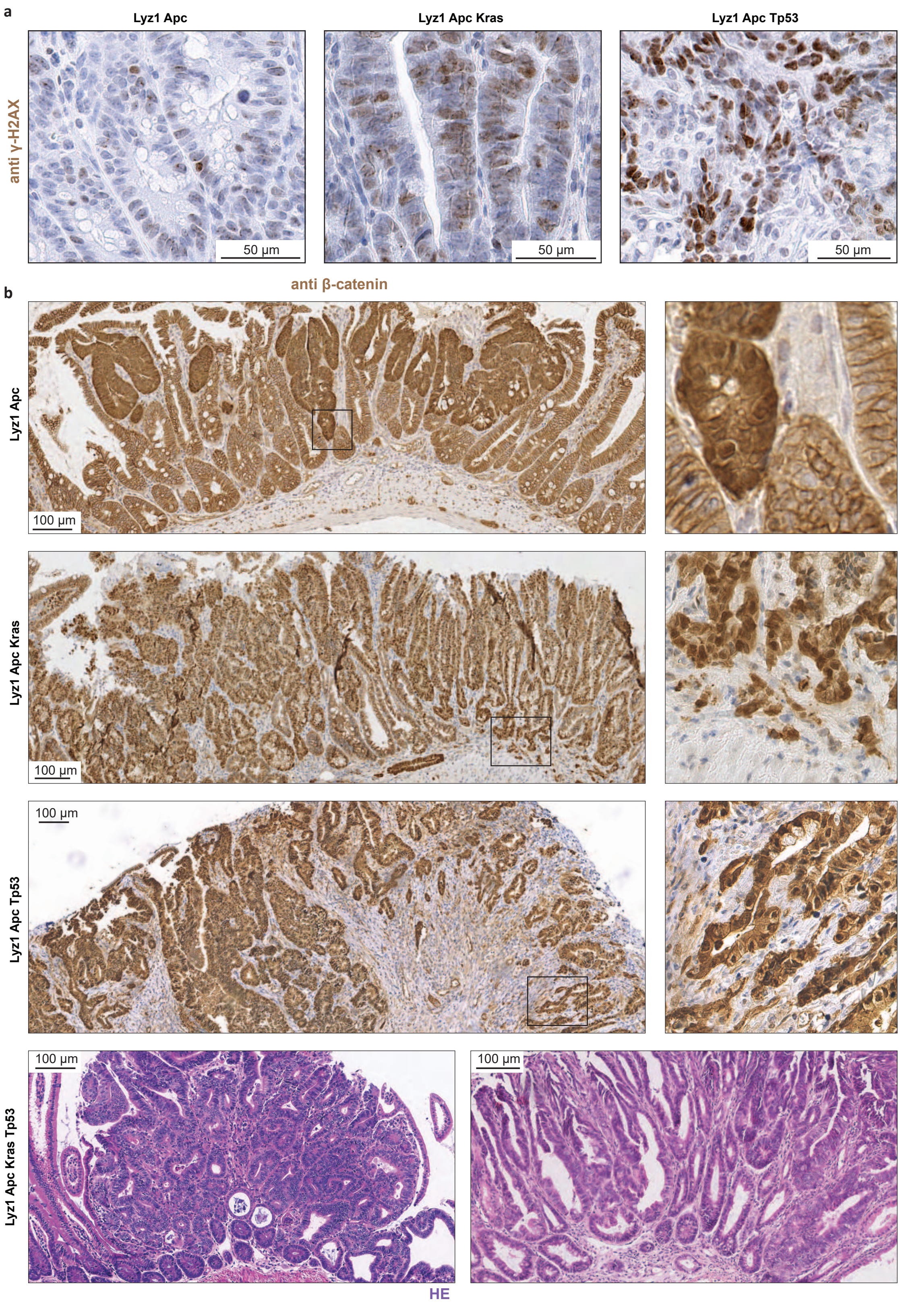

Suppl. Fig. 1 | Histological analysis of Paneth-derived tumors.

a. Representative IHC pictures of Paneth-derived tumors from different genetic backgrounds, stained for γ-H2AX.

b. Top: IHC analysis of Paneth-derived intestinal tumors with different genetic backgrounds, stained for β-catenin. Bottom: hematoxylin and eosin histological stains of Paneth-derived tumors from a Lyz1/Apc/Kras/Tp53 mouse.

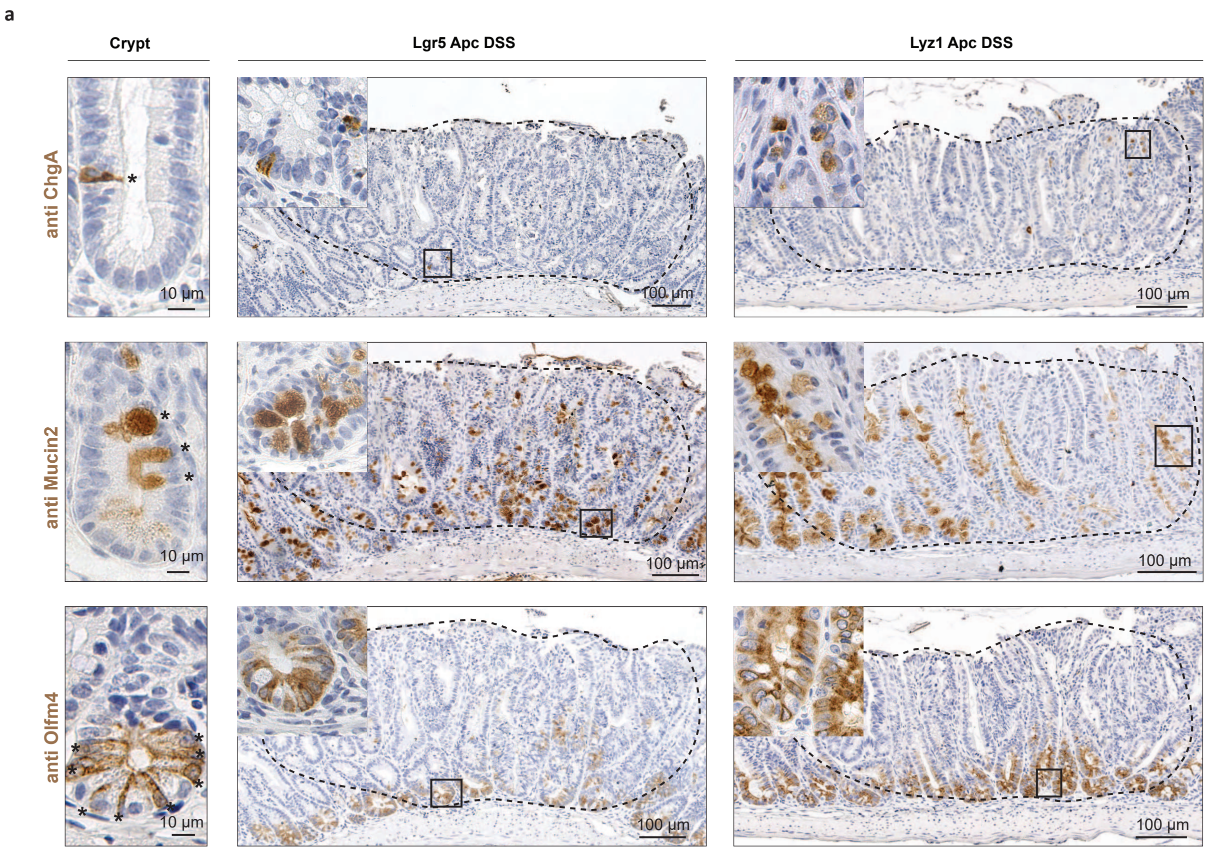

Suppl. Fig. 2 | Lineage-specific markers in Paneth and Lgr5-derived adenomas.

Representative IHC analyses of Paneth- and Lgr5-derived tumors, stained for Chga, Muc2, and Olfm4.

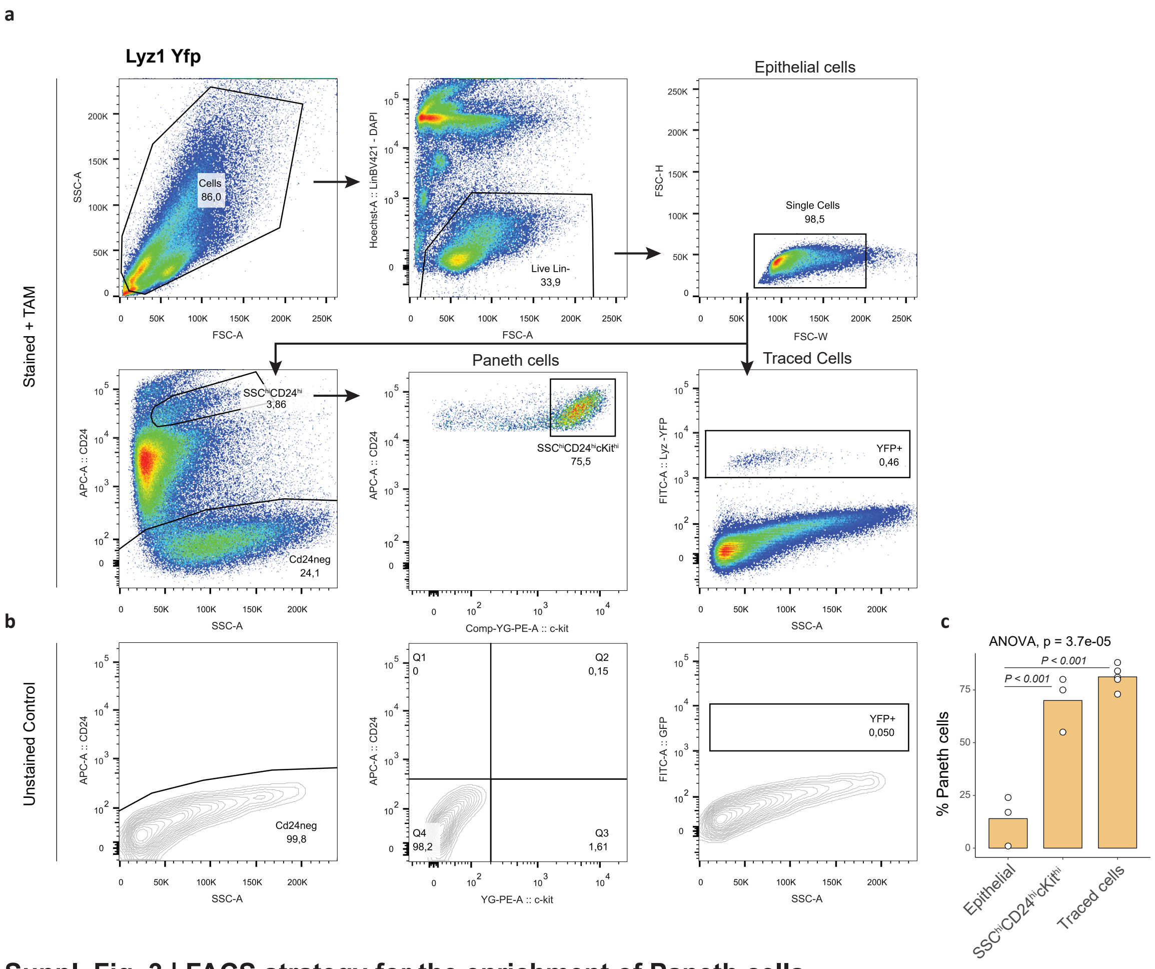

Suppl. Fig. 3 | FACS strategy for the enrichment of Paneth cells.

a. FACS gating strategy established for the isolation of live, single, epithelial cells (top). Subsequently, Paneth cells were enriched using the $SSC^{hi}CD24^{hi}$ gate and further purified based on high cKit levels ($SSC^{hi}CD24^{hi}cKit^{hi}$). Alternatively, traced cells were sorted based on positive expression for Yfp or td-Tomato. **b**. Negative control employed to establish reference levels based on unstained samples. **c**. Bar plot of the percentage of Paneth cells obtained by the distinct gating strategies. P values depict results of one-way ANOVA and Tukey tests.

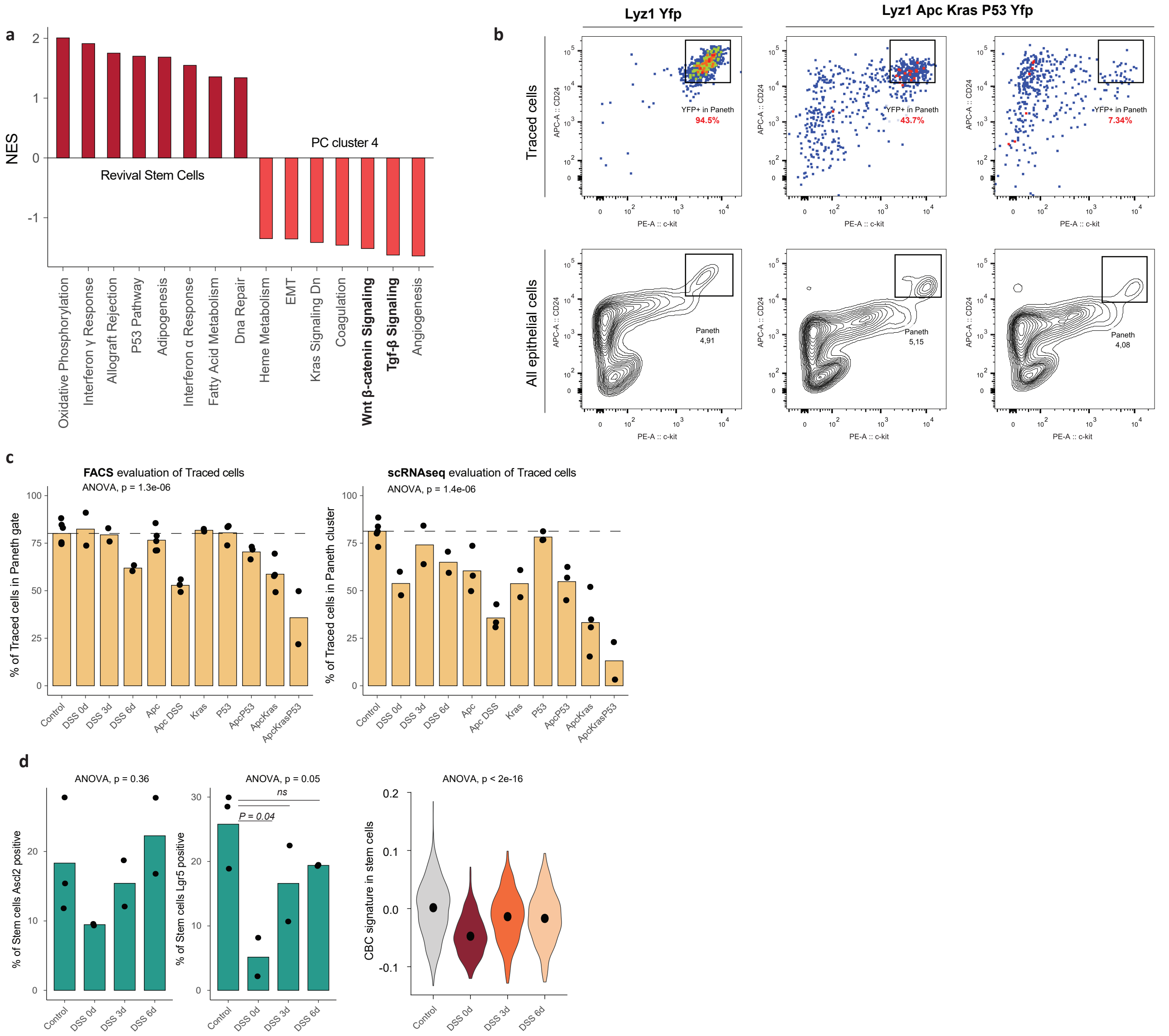

Suppl. Fig. 4 | Paneth cells dedifferentiate upon DSS administration or genetic targeting, while ISCs lose their multipotency.

a. Bar plot showing filtered pathways (Pval < 0.05, abs NES > 0.5) of the gene set enrichment analysis comparing Revival Stem Cells with PC Cluster 4. **b**. FACS plots showing how lineage-traced (Yfp+) cells localize outside of the CD24^{hi}cKit^{hi} gate upon genetic targeting. **c**. Bar plot relative to the percentages of lineage-traced cells that fall within the Paneth gate by FACS (SSC^{hi}CD24^{hi}cKit^{hi}; left), and after scRNAseq analysis (% within Paneth cluster; right). P values denote significance of one-way ANOVA. **d**. Left: Bar plots showing the average percentage of Ascl2^{+} and Lgr5^{+} stem cells in control and DSS-treated animals. Right: Violin plots showing a decrease in the crypt base columnar (CBC) signature upon DSS treatment. P values denote significance of one-way ANOVA and Tukey test for group comparisons.

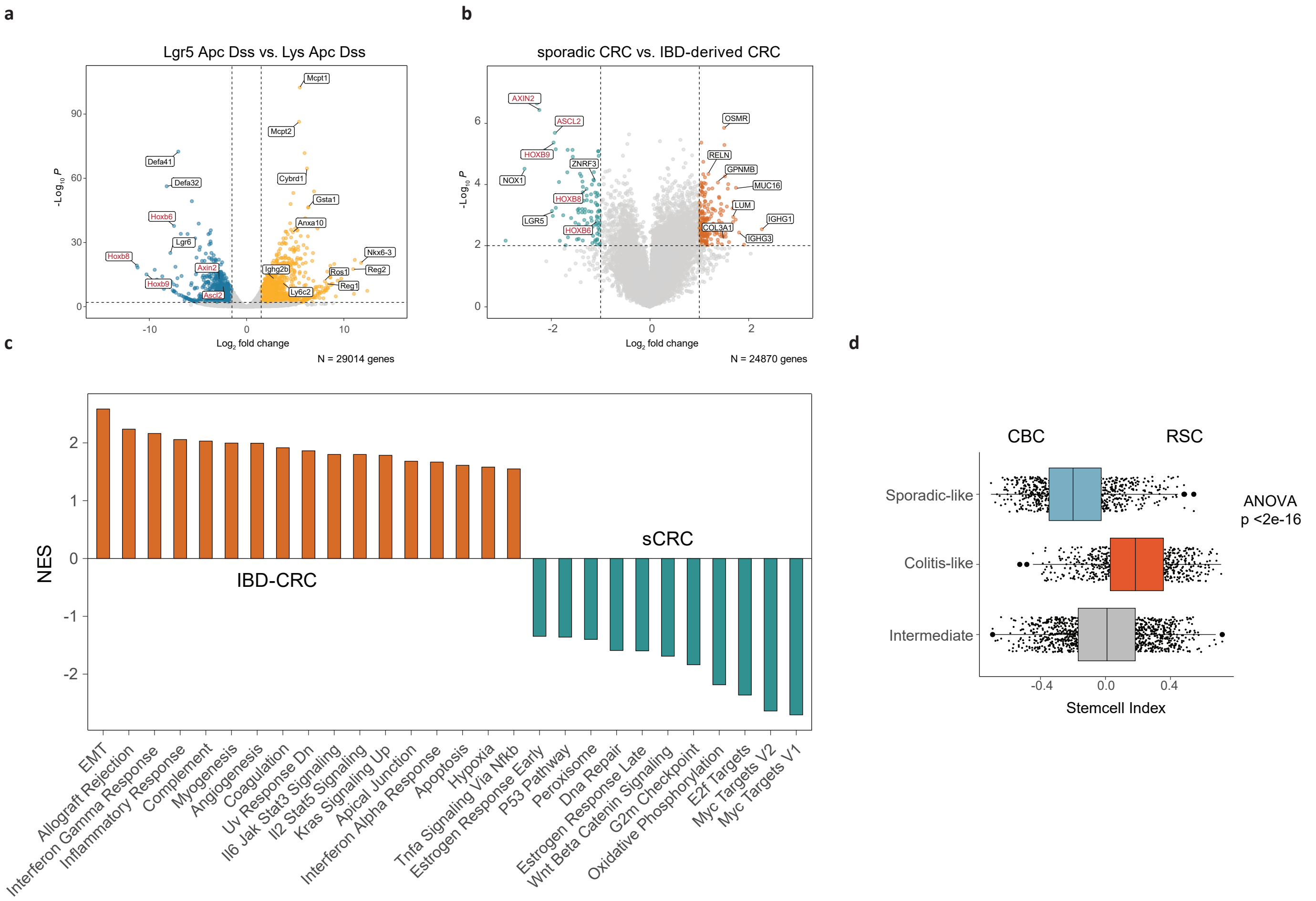

Suppl. Fig. 5 | Transcriptomic comparison between IBD-CRCs and murine Paneth-derived tumors.

a. Vulcano plot showing differentially expressed (Pval < 0.01, FCcutoff 1.5) genes between Lgr5-derived and Paneth-derived intestinal tumors. **b**. Vulcano plot showing differentially expressed (Pval < 0.01, FCcutoff 1) genes between sporadic- vs. IBD-CRCs. **c**. Bar plot showing the result of the gene set enrichment analysis comparing sCRC with IBD-CRC (abs NES > 0.5, Pval < 0.05). **d**. Box plots showing results of the stem cell index across the colitis-like, sporadic-like and intermediate group of colon cancers. P value depicts result of one-way ANOVA.

a

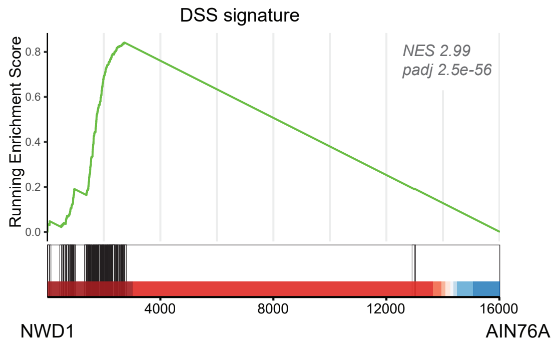

b

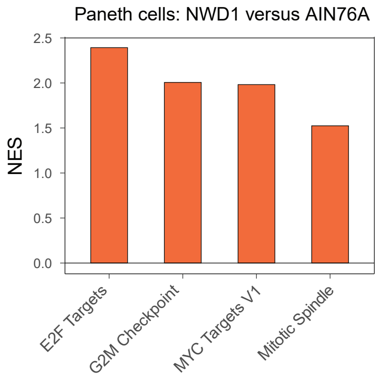

Suppl. Fig. 6 | Western-style diet triggers an inflammatory-like response in Paneth cells.

a. GSEA plot of Paneth cells from NWD1- vs. AIN76A-fed mice. The DSS signature significantly associates with the NWD1 diet (NES 2.99, Padj 2.5e-56). **b**. Bar plot showing pathways elevated ($P < 0.05$, NES > 0.5) in Paneth cells from NWD1-compared to AIN76A-fed animals.

Supplementary Files

This is a list of supplementary files associated with this preprint. Click to download.

- SupplementaryTable3.xlsx
- SupplementaryTable4.xlsx
- SupplementaryTable2.xlsx
- SupplementaryTable1.xlsx
